# Motives that Mediate the Associations Between Relationship Satisfaction, Orgasmic Difficulty, and the Frequency of Faking Orgasm

**DOI:** 10.1016/j.esxm.2022.100568

**Published:** 2022-09-14

**Authors:** Krisztina Hevesi, Zsolt Horvath, Eszter Miklos, Dorottya Sal, David L. Rowland

**Affiliations:** 1 Institute of Psychology, ELTE Eötvös Lorand University, Budapest, Hungary; 2 Department of Psychology, Valparaiso University, Valparaiso IN, USA

**Keywords:** Faking Orgasm, Pretending Orgasm, Women, Orgasmic Difficulty, Relationship Satisfaction

## Abstract

**Introduction:**

Faking orgasm by women reportedly occurs quite frequently, with both relationship characteristics and orgasmic difficulty being significant predictors.

**Aim:**

We explored women's motives that might mediate the associations between orgasmic difficulty and relationship satisfaction on the one hand, with the frequency of faking orgasm on the other.

**Methods:**

In a study of 360 Hungarian women who reported “ever” faking orgasm during partnered sex, we assessed the direct and indirect (mediated) associations between orgasmic difficulty, relationship satisfaction, and the frequency of faking orgasm.

**Outcomes:**

Determination of motives that mediate the association between orgasmic difficulty and the frequency of faking orgasm, and the association between relationship satisfaction and the frequency of faking orgasm.

**Results:**

Increased orgasmic difficulty was directly related to increased frequency of faking orgasm (β = 0.37; *P* < .001), and each variable itself was related to a number of motives for faking orgasm. However, the only motive assessed in our study that mediated the relationship between orgasmic difficulty and the frequency of faking orgasm was insecurity about being perceived as abnormal or dysfunctional (indirect effect: β = 0.13; *P* < .001). A similar pattern emerged with relationship satisfaction and frequency of faking orgasm. These two variables were directly related in that lower relationship satisfaction predicted higher frequency of faking orgasm (β = -0.15; *P* = .008). Furthermore, while each variable itself was related to a number of motives for faking orgasm, the only motive assessed in our study that mediated the relationship between the 2 variables was insecurity about being perceived as abnormal or dysfunctional (indirect effect: β = -0.06; *P* = .008).

**Clinical Translation:**

Insecurity related to being perceived as abnormal or deficient, along with sexual communication, should be addressed in women with a history of faking orgasm but who want to cease doing so.

**Strengths and Limitations:**

The sample was relatively large and the online survey adhered to best practices. Nevertheless, bias may result in sample characteristics when recruitment is achieved primarily through social media. In addition, the cross-sectional sample prevented causal determination and represented Western-based values.

**Conclusions:**

The associations between orgasmic difficulty and faking orgasm, and between relationship satisfaction and faking orgasm, are both direct and indirect (mediated). The primary motive for mediating the indirect association between the predictor variables and the frequency of faking orgasm was the insecurity about being perceived as deficient or abnormal.

**Hevesi K, Horvath Z, Miklos E, et al. Motives that Mediate the Associations Between Relationship Satisfaction, Orgasmic Difficulty, and the Frequency of Faking Orgasm. Sex Med 2022;10:100568.**

## INTRODUCTION

An estimated 30% to 75% of women have faked orgasm over their lifetime,^1^, ^2^, ^3^, ^4^, ^5^, ^6^, ^7^, ^8^ and this rate may be increasing among younger cohorts.^9^ Faking orgasm varies with a number of factors, including the woman's motivations^10^^,^^11^ and the type and characteristics of the dyadic relationship.^8^ Regarding the former, motivations for faking orgasm may be externally driven, that is, related to situational factors such as fatigue, boredom and/or wanting to end sex, and intoxication. Or they may be internally-driven, that is, related to the woman's momentary disposition or temperament, such as realizing that orgasm is unlikely or avoiding shame and embarrassment.^3^^,^^5^^,^^6^^,^^10^^,^^12^ Furthermore, internally-driven motivations may be either self-focused (as in avoiding embarrassment) or partner focused^7^ (as in maintaining the partner's interest and/or arousal and/or avoiding conflict and undesired conversation).^2^^,^^10^^,^^12^, ^13^, ^14^, ^15^, ^16^, ^17^, ^18^ Among partner-focused motivations, the desire to protect the partner's feelings and self-esteem appears to be one of the more common reasons for faking orgasm.^7^^,^^15^^,^^18^^,^^19^^,^^20^

Beyond motivations, faking orgasm also shows variation across types of relationship, not surprising given that women's motivations for being in specific types of relationships vary considerably.^21^^,^^22^ For example, casual relationships are presumably motivated more by the woman's own sexual pleasure and self-esteem, so faking orgasm for partner-focused reasons would be less likely.^23^ Yet, women in long-term relationship might also be less likely to fake orgasm, as motivations related to deception, boredom, or avoidance would typically prove counterproductive; that is, such women would presumably be more tolerant of the sometimes-disappointing sexual outcomes, given their commitment to the partner and the desire for a stable relationship. Research findings bear out the complex relationships between relationship type and faking orgasm: women in long-term relationships have been found to fake orgasm less^24^ than women in the general population, but they have also been found to fake orgasm more frequently, presumably out of concern for their partner.^8^ These disparate patterns are further complicated, as women in long-term relationships are also more likely to experience orgasm during partnered sex (67%)—which may decrease the pressure to fake orgasm—than women with a new partner (34%) or in a one-night stand (11%).^25^^,^^26^

## RELATIONSHIP TYPE, QUALITY, AND SATISFACTION

Attempting to understand variation in frequency of faking orgasm by focusing on relationship *type* undoubtedly oversimplifies the issue. Specifically, one-night stands and ongoing sexually-based relationships have typically been associated with lower rates of faking orgasm. Indeed, such patterns are plausible in one-night stands where a woman's right to pleasure is often not assumed by either the man or woman, and therefore the motivation for the woman to save face or please the partner by faking orgasm may be diminished.^19^ Similarly, ongoing *sexual* relationships are typically driven by the desire for sexual pleasure, and in such relationships, faking orgasm would miscommunicate important information to the partner related to her goal of sexual satisfaction.

In contrast with primarily sex-based relationships, ongoing romantic, stable, and long-term relationships are not easily characterized by a unified reason or motivation as they are, by definition, both complex and varied. It is within such relationships where substantial inconsistencies in faking orgasm appear to arise. For these relationships, the quality and satisfaction of the dyadic relationship—itself often varying with the developmental stage of the relationship—are likely more relevant to understanding and predicting orgasm faking than simply knowing the *type* of the relationship. For example, partner-focused motivations for faking orgasm—typically more common in stable, long-term relationships—may serve to protect and enhance the dyadic relationship by boosting the (male) partner's self-esteem or by stimulating the partner's interest and arousal.^1^^,^^5^^,^^8^^,^^15^^,^^16^^,^^27^^,^^28^ But they might also represent a strategy to avoid various negative repercussions, particularly in situations where the woman feels insecure about her relationship.^14^^,^^29^ By faking orgasm, the woman might avoid conflict or unpleasant conversation, or even discourage a wayward partner. According to several reports,^30^^,^^31^ since women's orgasm can contribute to men's sexual satisfaction, faking orgasm may be used not only to promote relationship stability, but also to counteract instability through partner retention strategies.^13^^,^^22^^,^^32^

Furthermore, the likelihood of a woman faking orgasm may diminish as the relationship matures, that is, as she feels increasingly assured of their partner's acceptance, develops confidence in herself, and/or becomes more satisfied with her sex life overall, regardless of reaching orgasm.^26^ Such findings suggest an inverse association between relationship quality and/or satisfaction and faking orgasm—as quality increases, orgasm faking tends to decrease.^24^ A similar association has been reported among Brazilian women, whose orgasm faking was used to avoid conflict and/or reinforce the partner's commitment in troubled relationships.^22^ Such disparate motives reiterate the point that faking orgasm in long-term relationships can involve both positive and negative motivations. On the one hand, it might indicate a lack of trust and intimacy within the relationship,^33^ but on the other hand, it could reflect a basic concern for the partner's self-esteem and enjoyment.^34^ To recapitulate then, because long-term relationships are often complex and varied—and typically change over time—the motivations for, and therefore the likelihood of, faking orgasm tend to show wide variation, being linked to relationship satisfaction and quality more than just relationship type.

## FAKING ORGASM AND ORGASMIC DIFFICULTY

A woman's intent to fake orgasm strongly suggests that she believes—for whatever reason—that orgasm will be unlikely during partnered sex. Thus, among the self-focused reasons for faking orgasm is the desire to avoid feelings of insecurity, shame, and deficiency associated with orgasmic difficulty (OD) during partnered sex.^5^^,^^7^^,^^10^^,^^14^^,^^16^^,^^35^, ^36^, ^37^, ^38^, ^39^ That is, difficulty reaching orgasm and the concomitant desire to disguise a perceived sexual deficiency may increase the likelihood of the woman's faking orgasm,^6^^,^^18^^,^^40^ with a number of studies documenting an association between faking orgasm and OD.^7^^,^^16^^,^^18^^,^^40^, ^41^, ^42^ Further supportive of this idea, OD has emerged as one of the strongest and most consistent predictors of the *frequency* of faking orgasm among women who reported “ever” faking an orgasm, independent of the type of relationship, including in romantic relationships that tend to change over time.^8^

## RATIONALE AND GOALS

As noted, both OD and relationship characteristics—type, quality, and satisfaction—are consistent predictors of the likelihood and frequency of faking orgasm.^8^^,^^15^^,^^35^, ^36^, ^37^ Yet, knowing about such associations reveals little about *why* women with greater OD or lower relationship satisfaction ultimately decide to fake orgasm. In this study on women who were currently in an ongoing romantic-sexual relationship, we explored the interrelationships among four variables—orgasmic problems, relationship satisfaction, women's motives for faking orgasm, and the frequency of faking orgasm—with the goal of identifying specific motives that might act as mediators between either OD or relationship satisfaction on the one hand, and faking orgasm on the other hand. We hypothesized:(i) That specific motives for faking orgasm would mediate the association between *orgasmic problems* and the frequency of faking orgasm. This relationship was investigated through three sub-hypotheses: (i) that higher levels of orgasmic problems would be associated with stronger motives for faking orgasm (eg, desireless sex, insecurity, partner self-esteem motives for faking orgasm), (ii) that higher frequency of faking orgasm would be positively associated with specific motives for faking orgasm, and (iii) that higher frequency of faking orgasm would be positively associated with higher rates of orgasmic problems.(ii) That specific motives for faking orgasm would mediate the association between *relationship satisfaction* and the frequency of faking orgasm. This relationship was investigated through three sub-hypotheses: (i) that lower relationship satisfaction would be associated with stronger motives for faking orgasm (eg, desireless sex, poor sex and/or partner, partner self-esteem motives for faking orgasm), (ii) that higher frequency of faking orgasm would be positively associated with specific motives for faking orgasm, and (iii) that higher frequency of faking orgasm would be positively associated with lower relationship satisfaction.^6^^,^^8^^,^^11^^,^^22^^,^^33^^,^^34^

## MATERIALS AND METHODS

### Participants and Procedures

In this cross-sectional study, women from Hungary completed an anonymous online questionnaire that included items about demographic, medical, and sexual history; sexual response and orgasmic functioning during partnered sex and masturbation; and the frequency of and motives for faking orgasm. Participants were recruited using three strategies: (i) invitations to local university students as one means to earn extra credit in their courses; (ii) postings on Facebook (social media site); and (iii) invitations via articles on sexual psychological themes in online Hungarian magazines. The Research Ethics Committee of the Faculty of Education and Psychology of the ELTE Eötvös Loránd University approved the study protocol (reference no. 2018/180). Before accessing the questionnaire, participants gave informed consent, declared being at least 18 years old, and acknowledged the voluntary and anonymous nature of the study as well as the option to terminate participation at any time without consequences.^8^

Overall, 2,200 individuals responded to the survey. However, to address the aims of the current study, only heterosexual, cisgender women, who had “ever” masturbated in their lifetime, who were in a current ongoing relationship with a sexual partner (ie, ever had a sexual intercourse and/or performed other sexual activities with the partner, such as anal or oral sex), and who reported “ever” faking orgasm during a sexual act with the partner, were included in the analyses. Of the 874 women currently in a relationship and actively having sex with their partner, 360 (41%) indicated that they had “ever” faked orgasm during (any type of) sex with their partner, having a mean age 32.58 years (SD = 10.08), and a mean relationship length was 7.73 years (SD = 7.90)

### Measures

Four major sets of variables were assessed in this study, those assessing: (i) relationship satisfaction; (ii) orgasmic difficulty and/or problems; (iii) the frequency of faking orgasm; and (iv) motives for faking orgasm, this last set of variables explored as potential mediating variables for understanding relationships among the first three variables. For specification of the overall model, refer to the section on “Statistical Model and Data Analysis.”

*Relationship satisfaction.* Relationship satisfaction was assessed by the Relationship Assessment Scale.^43^ Originally, this scale contained 7 items measuring the general level of relationship satisfaction. In the present study, a modified, 8 item version included an additional item on sexual relationship satisfaction.^44^ Participants responded on a 5 point scale (1 = characteristic only a little, 5 = very characteristic). A high level of internal consistency was found for the scale as used in the current study (Table 1).

**Table 1:** Bivariate correlations and descriptive statistics for the four major sets of variables

	1.	2.	3.	4.	5.	6.	7.	8.	9.	10.	11.	12.	13.
1. Length of relationship	-	-0.21	-0.30*	-0.06	-0.10	-0.13	0.22*	0.01	-0.02	-0.03	0.09	0.03	0.09
2. Relationship satisfaction		-	0.42*	-0.36*	-0.15	0.05	-0.35*	-0.26*	-0.17	0.00	-0.49*	0.00	-0.39*
3. Frequency of sex			-	-0.14	-0.04	0.13	-0.20*	-0.02	0.10	0.05	-0.14	0.05	-0.16
4. Orgasmic problems in partnered sex				-	0.14	0.16	0.15	0.40*	0.10	0.20*	0.26*	0.05	0.58*
5. Frequency of masturbation					-	-0.22*	-0.04	0.12	0.06	-0.03	0.08	-0.01	0.11
6. Orgasmic problems in masturbation						-	-0.04	0.03	0.02	0.11*	0.03	-0.03	-0.01
7. Desireless sex motive for faking orgasm							-	0.23*	0.17	0.11	0.52*	0.13	0.26*
8. Insecurity motive for faking orgasm								-	0.24	0.46*	0.42*	0.36*	0.54*
9. Intoxication motive for faking orgasm									-	0.01	0.33*	0.08	0.13*
10. Partner self-esteem motive for faking orgasm										-	0.13	0.33*	0.29*
11. Poor sex/partner motive for faking orgasm											-	0.13	0.31*
12. Timing motive for faking orgasm												-	0.16*
13. Frequency of faking orgasm													-
M (SD)	7.73 (7.90)	31.76 (5.97)	6.45 (1.36)	0.00 (1.00)	4.95 (1.87)	0.00 (1.00)	12.90 (6.53)	16.92 (8.98)	4.39 (3.29)	26.69 (7.64)	7.03 (4.82)	12.82 (6.50)	0.00 (1.00)
Cronbach's α	-	0.89	-	0.83	-	0.82	0.73	0.78	0.88	0.85	0.77	0.93	0.88

Note. N = 325-360. Correlation estimates were calculated by using the maximum likelihood robust to non-normality (MLR) estimation method.

⁎ In order to control for family-wise Type I error, correlation estimates were considered significant only if they reached the *P* < .001 level (highlighted in bold).

*Orgasmic difficulty and/or problems in partnered sex and masturbation.* Orgasmic response/problems was measured by specific items from a 42 item questionnaire.^42^^,^^45^, ^46^, ^47^ Participants’ orgasmic response was assessed through eight items, four related to partnered sex and four parallel items related to masturbation. These included: (i) frequency of reaching orgasm (ie, the estimated proportion of occasions reaching orgasm relative to the overall number of sexual episodes; 1 = Never, 10 = Always), (ii) difficulty reaching orgasm (0 = Always reaching orgasm, 5 = Nearly always [having difficulty reaching orgasm]), (iii) orgasmic latency (1 = 1−5 minutes [to reach orgasm], 7 = I do not reach orgasm), and (iv) lack of orgasmic pleasure (1 = Very satisfying and/or pleasurable, 6 = Do not reach orgasm).

Principal component analysis (PCA) was applied to create separate composite measures for orgasmic problems during partnered sex (explained variance by the component: 70.59%) and during masturbation (explained variance by the component: 75.58%). High correlations among the variables justified the construction of the composite variables (partnered sex:|*r*|=0.54–0.64; masturbation: |*r*|=0.52–0.70). Previous studies have used this same strategy successfully in the study of women's orgasmic problems during partnered sex and masturbation.^45^, ^46^, ^47^ High levels of internal consistency were shown for these two composite measures of orgasmic problems (Table 1).

In addition to these measures of orgasmic response, participants estimated, on two separate questions, their frequency of partnered sex and their frequency of masturbation during the past 9-12 months (1 = Almost never, 8 = One or more times daily).

*Frequency of faking orgasm.* Frequency of faking orgasm within the current relationship was evaluated in four contexts using four separate items.^8^^,^^14^ Participants rated on an 11 point scale (0 = Never, 10 = Always), (i) how frequently they faked orgasm during a sexual intercourse, and (ii) other sexual activities not involving vaginal or anal penetration; and (iii) how frequently they faked being more sexually excited than they really were during sexual intercourse, and (iv) other sexual activities not involving vaginal or anal penetration. Due to the reasonably high correlations among the four frequency measures (*r* = 0.59–0.71), a composite variable was constructed by using PCA to measure the overall frequency of faking orgasm in the current relationship. A satisfactory level of internal consistency was found for this composite measure (Table 1).

*Motives for faking orgasm.* The Motives for Feigning Orgasms Scale (MFOS) was used to assess motives for faking orgasm specifically within the context of the current relationship.^48^ The 25 item questionnaire contains six different motives for faking orgasm: (i) desireless sex (eg, felt tired or wanted to sleep), (ii) insecurity (eg, wanted to avoid appearing abnormal or inadequate), (iii) intoxication (eg, had too much drink), (iv) partner self-esteem (eg, wanted to make partner feel good about himself), (v) poor sex and/or partner (eg, felt uncomfortable with partner), and (vi) timing (eg, partner not ready to have an orgasm). Participants provided responses on a 7 point scale (1 = not at all important, 7 = extremely important). Subscales of the questionnaire demonstrated moderate-high levels of internal consistency (Table 1).

### Statistical Model and Data Analysis

As an initial step, bivariate correlations were calculated between the study variables by using the maximum likelihood robust to non-normality (MLR) estimation method. Correlations were considered significant at *P* < .001 in order to control for family-wise Type I error.

Next, a linear regression-based mediation model was proposed and tested, with the two aims of the present study investigated within a single mediation model. All variables in the model were specified as observed variables. Orgasmic problems in partnered sex and masturbation, and relationship satisfaction were distal predictor variables. Six mediator variables were included in the model, all of which measured motives for faking orgasm: desireless sex, insecurity, intoxication, partner self-esteem, poor sex and/or partner, timing motives for faking orgasm. The overall frequency of faking orgasm was the outcome variable of the mediation model. Moreover, the covariate effects of length of relationship and frequency of partnered sex and masturbation were also controlled in the analysis. Indirect effect size estimates were calculated on the relationships between orgasmic problems in partnered sex and masturbation, relationship satisfaction, and frequency of faking orgasm via motives for faking orgasm. The MLR estimation method was applied to calculate parameters in the mediation model. Mplus 8.0^49^ and SPSS (SPSS Statistics for Windows, version 25, SPSS Inc., Chicago, Ill., USA) were used to perform the analyses.

## RESULTS

### Pairwise Correlations

Bivariate correlations and descriptive statistics are given in Table 1. Presentation of correlations are organized according to three major variables: relationship satisfaction, orgasmic problems in partnered sex and masturbation, and motives for faking orgasm.

Higher relationship satisfaction was significantly and weakly-moderately linked to more frequent partnered sex, and lower levels of all the following: orgasmic problems in partnered sex; desireless sex, insecurity, and poor sex and/or partner motives for faking orgasm; and frequency of faking orgasm.

Elevated rates of orgasmic problems in partnered sex were significantly associated with higher insecurity, partner self-esteem, and poor sex and/or partner motives for faking orgasm, and frequency of faking orgasm. Orgasmic problems in masturbation had a significant, weak and negative correlation with frequency of masturbation.

Regarding the various motives for faking orgasm, higher rates of desireless sex motive for faking orgasm were significantly and weakly linked to longer relationship length, less frequent partnered sex and were significantly and weakly associated with higher levels insecurity motive for faking orgasm and frequency of faking orgasm. Insecurity motive for faking orgasm presented significant, positive and moderate-strong correlations with partner self-esteem, poor sex and/or partner and timing motives for faking orgasm and frequency of faking orgasm. Higher levels of poor sex/partner motive were significantly, positively and moderately-strongly associated with desireless sex and intoxication motives for faking orgasm and frequency of faking orgasm. Partner self-esteem motive for faking orgasm had significant, positive and weak-moderate correlations with timing motive for faking orgasm and frequency of faking orgasm.

### Aim 1: Motives Mediating the Association Between Orgasmic Problems and Faking Frequency

**Figure 1:**
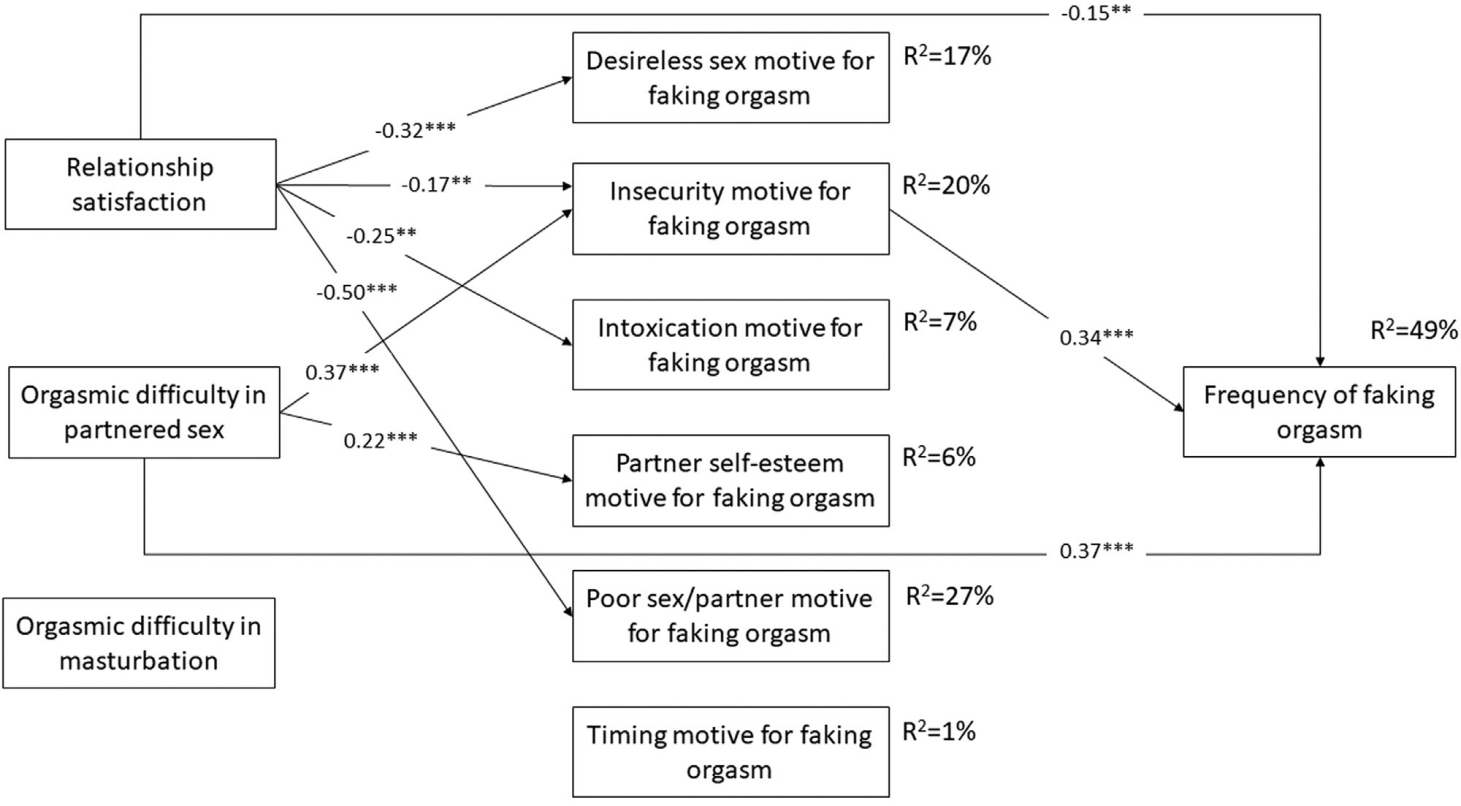
Significant predictive effects in the mediation model. Notes. N = 349. Values in each arrow are standardized regression coefficients (β). Level of significance: *P < .050; **P < .010; ***P < .001. Non-significant (*P* > .050) regression coefficients and covariates (ie, frequency of sex and masturbation, length of relationship) are not shown in the figure to ease the interpretation (see further: Table 2).

Standardized regression coefficients representing the predictive effects in the mediation model for Aim 1 are shown in Table 2 and Figure 1. Higher levels of orgasmic problems during partnered sex were significantly and positively associated with higher rates of the insecurity and partner self-esteem motives for faking orgasm. Moreover, higher frequency of faking orgasm was significantly and positively associated with orgasmic problems during partnered sex and the insecurity motive for faking orgasm.

**Table 2:** Predictive effects in the mediation model

Predictor variables	Mediator variables						Outcome variable
	Desireless sex motive for faking orgasm	Insecurity motive for faking orgasm	Intoxication motive for faking orgasm	Partner self-esteem motive for faking orgasm	Poor sex/partner motive for faking orgasm	Timing motive for faking orgasm	Frequency of faking orgasm
	β (S.E.)	β (S.E.)	β (S.E.)	β (S.E.)	β (S.E.)	β (S.E.)	β (S.E.)
Length of relationship	0.16 (0.05)⁎⁎	0.05 (0.06)	0.00 (0.06)	0.00 (0.07)	0.04 (0.05)	0.06 (0.06)	0.05 (0.04)
Relationship satisfaction	-0.32 (0.06)⁎⁎⁎	-0.17 (0.06) ⁎⁎	-0.25 (0.08) ⁎⁎	0.05 (0.07)	-0.50 (0.06) ⁎⁎⁎	-0.02 (0.06)	-0.15 (0.06) ⁎⁎
Frequency of sex	-0.01 (0.06)	0.13 (0.06)*	0.20 (0.05) ⁎⁎⁎	0.07 (0.06)	0.10 (0.06)	0.10 (0.06)	-0.01 (0.04)
Orgasmic problems in partnered sex	0.07 (0.06)	0.37 (0.06) ⁎⁎⁎	0.05 (0.06)	0.22 (0.05) ⁎⁎⁎	0.11 (0.06)	0.06 (0.06)	0.37 (0.06) ⁎⁎⁎
Frequency of masturbation	-0.10 (0.06)	0.04 (0.06)	0.02 (0.06)	-0.05 (0.06)	-0.01 (0.05)	-0.03 (0.06)	0.00 (0.04)
Orgasmic problems in masturbation	-0.04 (0.06)	-0.03 (0.05)	0.00 (0.06)	0.05 (0.05)	0.03 (0.06)	-0.06 (0.06)	-0.07 (0.04)
Desireless sex motive for faking orgasm	-	-	-	-	-	-	0.08 (0.05)
Insecurity motive for faking orgasm	-	-	-	-	-	-	0.34 (0.06) ⁎⁎⁎
Intoxication motive for faking orgasm	-	-	-	-	-	-	-0.02 (0.05)
Partner self-esteem motive for faking orgasm	-	-	-	-	-	-	0.06 (0.05)
Poor sex/partner motive for faking orgasm	-	-	-	-	-	-	-0.04 (0.07)
Timing motive for faking orgasm	-	-	-	-	-	-	0.00 (0.04)
Explained variance (R^2^)	17%	20%	7%	6%	27%	1%	49%

N = 349. In each cell standardized regression coefficients (β) and standard error (S.E.) values are shown. Correlations between the mediator variables were estimated, but they are not reported in the table to ease the interpretation. Range of correlations between the mediator variables: *r* = -0.01 (between Intoxication and Partner self-esteem motives) – 0.46 (between Insecurity and Partner self-esteem motives); mean of correlations between the mediator variables: *r* = .22.

⁎ *P* < .050;

⁎⁎ *P* < .010;

⁎⁎⁎ *P* < .001.

Regarding direct and indirect effects, the effect of orgasmic problems during partnered sex on the frequency of faking orgasm remained significant over the effects of the mediator variables, indicating a direct effect between orgasmic problems and the frequency of faking orgasm. Moreover, a significant indirect effect was also identified related to Aim 1 in that higher levels of orgasmic problems in partnered sex contributed to higher levels of the insecurity motive for faking orgasm which, in turn, predicted more frequent faking orgasm presence (β (S.E.) = 0.13 (0.03) *P* < .001) (not seen in the table). Thus, both direct and indirect effects were evident in the relationship between orgasmic problems during partnered sex and the frequency of faking orgasm. None of the associations with orgasmic problems in masturbation were significant in the mediation model.

### Aim 2: Motives Mediating the Association between Relationship Satisfaction and Faking Frequency

Predictive effects regarding Aim 2 are also shown in Table 2 and Figure 1. Lower relationship satisfaction was significantly linked to higher levels of the desireless sex, insecurity, intoxication, and poor sex/partner motives for faking orgasm. Higher frequency of faking orgasm was significantly linked to lower relationship satisfaction and higher levels of insecurity motive for faking orgasm.

Regarding direct and indirect effects, the effect of relationship satisfaction on the frequency of faking orgasm remained significant over the effects of the mediator variables, indicating a direct effect between lower relationship satisfaction and a higher frequency of faking orgasm. However, a significant indirect effect was also identified in that decreased relationship satisfaction was associated with higher level of insecurity motive for faking orgasm, which was subsequently related to a higher frequency of faking orgasm (β (S.E.) = -0.06 (0.02) *P* = .008).

## DISCUSSION

In this study, we were able to identify specific motives that act as mediators for faking orgasm. First, we specified mediating motives to explain the association between greater orgasmic difficulty and higher frequency of faking orgasm, and second to explain the association between lower relationship satisfaction and higher frequency of faking orgasm.

### Orgasmic Difficulty and Faking Orgasm: Direct and Mediating Factors

Regarding the relationship between orgasmic difficulty and faking orgasm, two pathways were identified: greater orgasmic difficulty on its own explained increased frequency of faking orgasm; at the same time, orgasmic difficulty also affected faking frequency through the woman's insecurity related to her orgasmic problems. In addition, women experiencing greater orgasmic problems were also more concerned about how it might affect their partner's self-esteem. Yet because this latter variable was itself not significantly related to orgasm faking, this analysis concludes that although orgasmic difficulty in women is related to both higher levels of insecurity *and* concern about their partner's self-esteem, it was the former motive—derived from their own feelings of insecurity—that was most strongly associated with their greater likelihood of faking orgasm. In other words, they faked orgasm because they were more concerned about being perceived as abnormal or deficient than they were about their partner's sense of satisfaction and/or esteem. This conclusion—demonstrated empirically in this analysis—is supported by a number of other studies that have argued that women may fake orgasm as a way of concealing their orgasmic difficulty, as well as avoiding the associated feelings of shame, embarrassment, and failure during partnered sex.^5^^,^^7^^,^^14^^,^^18^^,^^38^, ^39^, ^40^ Such a pattern of faking orgasm might be expected in short term or early-stage relationships,^8^ but we were actually quite surprised to find this pattern in women in ongoing relationships. Specifically, we had assumed that issues regarding women's orgasmic difficulty would have become part of the sexual communication that typically emerges in couples as the relationship matures (eg,^24^^,^^26^) and further, that the need to consistently reach orgasm during partnered sex would no longer be viewed as a *sine qua non* for a fulfilling sexual interaction.^26^ Such an assumption on the part of the woman and her partner would, in the longer term, counter the woman's perception that lack of orgasm during partnered sex reflects some sort of deficiency.

Despite such assumptions, in our sample of women who faked orgasm, motivations were more self-focused than partner-focused. It may be, of course, that because men's orgasm may take preference over women's orgasm in some relationships, the woman was focused more on her own pleasure than that of a potentially satisfied partner.^8^^,^^21^, ^22^, ^23^ Furthermore, it may be that the woman's “insecurity” was strongly tied to a general anxiety surrounding sexual activity, a situational variable that the woman may have felt was temporarily interfering with her capacity to reach orgasm,^50^^,^^51^ but one that was not assessed in our study.

We do note, however, that women's concern for the partner's self-esteem was significantly correlated with their own feelings of insecurity. In this regard, women who fake orgasms in their ongoing relationship generally may show greater sensitivity—and perhaps vulnerability—to the perception of others, including their partner. Such individuals may be more self-critical and empathic, with a tendency toward people-pleasing.^34^

Equally interesting are the mediating pathways that appear to be less or not important between orgasmic difficulty and orgasm frequency. Specifically, poor or desireless sex and having negative partner issues did not emerge as relevant factors in this pathway. In our sample, those women who were experiencing relational issues were generally those in longer relationships, and we surmise they generally had less reason/incentive to fake orgasm—they may not only have been less concerned about their partner's response and/or feelings, but they may well have sought sexual satisfaction through other outlets such as masturbation.^50^^,^^51^ Furthermore, these women did not appear to respond with orgasm faking to situations over which they had little influence, for example, being intoxicated or a partner who was taking too long to reach orgasm.

### Relationship Satisfaction and Faking Orgasm: Direct and Mediating Factors

The associations between relationship satisfaction and faking orgasm somewhat echoed those of orgasmic difficulty. Lower relationship satisfaction was directly associated with a higher frequency of faking orgasm. It was also associated with the desireless sex motive, partner-related motives, the intoxication motive, and the insecurity motive; but only the insecurity motive served as a mediating variable between relationship satisfaction and a higher frequency of faking orgasm.

Thus, poorer relationship satisfaction increased faking frequency either directly—an association iterated in prior research^5^^,^^8^^,^^15^^,^^16^^,^^27^, ^28^, ^29^—or indirectly via the insecurity motive or some other motive and/or factor not assessed in our study. Given that orgasmic problems and relationship satisfaction were themselves correlated (13% covariance) and that both were directly associated with the insecurity motive, we invoke a similar explanation for the mediating role of the insecurity motive between relationship satisfaction and faking frequency as was proposed for the relationship between orgasmic difficulty and faking frequency. Specifically, the lower the woman's relationship satisfaction, the greater her insecurity about herself—likely a partial proxy for sexual anxiety—and the greater her frequency of faking orgasm.^3^^,^^5^^,^^8^, ^9^, ^10^^,^^52^

In contrast, although lower relationship satisfaction was robustly linked to desireless sex and poor sex/partner motives (eg, feeling distant or uncomfortable with the partner), these conditions *per se* did not generally lead to greater frequency of faking orgasm. Women reporting lower relationship satisfaction may have been so minimally invested in their sexual and/or overall relationship that that they felt no need to pretend to be sexually satisfied—whether for their own self-esteem or out of concern for their partner.

It is well known that the *context* of the woman's relationship likely influences her perceived pressure to fake orgasm. For example, men in longer-term and/or committed relationships attend more to pleasing their partners, with relationships characterized by mutual care and commitment typically involving a greater variety of sexual activities and thereby increasing the woman's likelihood of orgasm^19^ and lessening the need to fake it. Thus, as a relationship matures, partner considerations typically assume greater importance, with such concerns as protecting the partner's self-esteem, avoiding partner disappointment, maintaining partner interest, and avoiding conflict and unpleasant conversations playing a greater role in motivating orgasm faking.^1^^,^^7^^,^^9^^,^^11^^,^^13^, ^14^, ^15^^,^^20^^,^^21^ One study, for example, reported that 47% of respondents faked orgasm to please their partner and 78% to avoid conflict or to spare their partner's feelings.^10^ Another has shown that faking orgasm is closely tied to the woman's assessment of the importance of her orgasm to her (male) partner as well as her worries about the effects of her lack of orgasm on him.^22^^,^^23^

However, our results are not entirely consistent with the line of thinking that greater relationship maturity and/or age is associated with stronger partner-focused motives for faking orgasm. To reiterate, our sample included a select group of women who identified themselves as being in an ongoing relationship *and* who reported “ever” faking orgasm during sex with their partner. In these women, whose average age was nearly 33 years and whose average relationship was close to 8 years, relationship length was related to only one motivation for faking orgasm, namely desireless sex. Most noticeably absent was any correlation between greater relationship length and increased concern about the partner's feelings or self-esteem. Indeed, in some long-term relationships, the opposite may occur, such that as the relationship settles in, the desire or pressure to please the partner diminishes.

### General Observations and Future Research

Our study identifies clear areas where future research could yield helpful information regarding orgasm faking. Specifically, knowing more about the developmental stage of the couple's relationship (rather than simply its duration or type) could be useful in understanding the evolution (and in some instances, de-evolution) of faking orgasm within an ongoing romantic relationship. Relationships undergo significant transition over time, with a number of authors^53^^,^^54^ identifying sequential phases that are sometimes progressive, at other times regressive. Given that relationship characteristics are known to influence orgasm faking, assessment of the couples “relationship phase” could help identify when and where women feel the greatest need (or desire) to fake orgasm during partnered sex. Indeed, a cost and/or benefit analysis might provide an interesting framework for understanding variation in orgasm faking during these different relationship phases.

Second, with the relatively high prevalence of faking orgasm among women, there is, in our view, need to assess men's response to perceived orgasm faking in women. One study, for example, has indicated that couples within a sexual relationship tend to be fairly astute regarding the sexual satisfaction of their partner.^55^ Yet, the high prevalence of orgasm faking would suggest that women believe they can effectively conceal the “faking” part of their orgasm from their partner. Are such men indeed oblivious about the behavior? Aware of the behavior yet complicit with and tolerant of the intended deception? Comfortable with the deception because it serves their needs of increased arousal and/or decreased guilt? Or distressed by the suspicion that such deception is likely occurring yet finding it too unpleasant to broach the topic? Qualitative and focus-group analysis would, in our view, help shed light on the alignment between the motives behind the woman's orgasm faking and the perceived effects on the (typically male) partner.

Finally, given that orgasm faking during partnered sex in women tends to provide affirmation of masculinity and male self-esteem, we pose the question as to whether similar rates and motives might exist within lesbian couples where, presumably, the masculinity issue is diminished or absent.

### Strengths and Limitations

Our study included the benefits common to many online and/or non-online surveys,^56^ including a sizable sample drawn from Hungary. In addition, we adhered to best practices in online survey construction, implementation, and distribution, ensuring participants of anonymity, embedding attention checks in the survey, implementing safeguards against multiple submissions from the same individual, and not offering rewards or incentives, thereby removing external motivators as a reason for survey completion.^56^, ^57^, ^58^, ^59^, ^60^, ^61^ At the same time, our conclusions were limited by the potential for systematic bias stemming from the recruitment process, a problem for any non-probability study that includes social media as one recruitment strategy. Furthermore, as we note in our discussion of the mediational effects of various motives, sexual distress and/or anxiety may have played an important role in faking orgasm, and although we had included a general measure of ongoing anxiety in our study, it would have been beneficial to assess situation-specific sexual distress and/or anxiety. We further note that the women in our sample predominantly represented Western-based values and assumptions, and therefore our findings may not be applicable to other world geo-cultural regions. Finally, due to the cross-sectional nature of the study, although we were able to specify sequential effects of relationships, we could not assume causality among these variables.

## CONCLUSIONS

Direct relationships between relationship satisfaction, orgasmic difficulty, and faking orgasm were identified in this analysis of women in longer-term, romantic-sexual relationships. The motive most clearly mediating the indirect association between both relationship satisfaction and orgasmic difficulty on the one hand, and the frequency of faking orgasm on the other, were the woman's feelings of insecurity. Future research might assess the developmental phase of the dyadic relationship and tap into the (male or female) partner's perceptions (or lack thereof) of the woman's orgasm faking behavior.

### Datafile Access

Interested researchers may make reasonable requests to review the output files from the analyses.

## Statement of Authorship

Krisztina Hevesi: Conceptualization, Methodology, Data Collection and Curation, Formal Analysis, Writing—Review and Editing; Zsolt Horvath: Methodology, Data Collection and Curation, Formal Analysis, Writing—Original Draft Preparation, Writing—Review And Editing; Eszter Miklos: Methodology, Data Collection and Curation, Investigation and Literature Review, Writing—Original Draft Preparation, Writing—Review and Editing; Dorottya Sal: Data Collection and Curation, Investigation and Literature Review, Writing—Original Draft Preparation, Writing—Review and Editing; David L. Rowland: Conceptualization, Methodology, Data Collection and Curation, Formal Analysis, Investigation and Literature Review, Writing—Original Draft Preparation, Writing—Review and Editing. All authors have read and agreed to the published version of the manuscript.
